# Pharmacokinetics of drugs used to treat drug sensitive-tuberculosis in breastfeeding mother-infant pairs: An observational pharmacokinetic study

**DOI:** 10.12688/wellcomeopenres.19113.3

**Published:** 2024-05-21

**Authors:** Ritah Nakijoba, Aida Nakayiwa Kawuma, Francis Williams Ojara, Jovia C. Tabwenda, Jacqueline Kyeyune, Christine Turyamureba, Simon Peter Asiimwe, Johnson Magoola, Barbara Castelnuovo, Allan Buzibye, Catriona Waitt

**Affiliations:** 1 Infectious Diseases Institute, College of Health Sciences, Makerere University, Kampala, Central Region, 256, Uganda; 2 Department of Pharmacology, Gulu University, Gulu, Northern Region, 256, Uganda; 3 Department of Pharmacology and Therapeutics, University of Liverpool, Liverpool, England, UK

**Keywords:** Tuberculosis, breastmilk pharmacokinetics, Africa, lactation

## Abstract

**Background:**

Globally, more than half of women take medicines whilst breastfeeding. Data concerning the exposure of the breastfed infant to drugs and any related risks are sparce. Lactation studies are only rarely performed close to licensing for medicines anticipated to be widely used in women of childbearing age. Medicines taken by breastfeeding mothers on tuberculosis (TB) treatment can be transferred to the breastfed infant. Potential effects of anti-tuberculosis medicines on nursing infants are not well understood. Similarly, women face mental health challenges while taking medications, including postpartum depression, hence the need to assess the psychological behavior of a breastfeeding woman. Potential risks are the development of adverse drug effects in the breastfed infant and selection for resistance, whereas potential benefits might include exposure to potentially prophylactic concentrations of the drug. Pharmacokinetic studies are therefore necessary to understand this situation fully.

**Methods:**

This study will enroll 20 mothers receiving first-line anti-tuberculosis medicines, together with their breastfed infants, with the aim of characterizing the breastmilk transfer of the medicines from the mother to the infants. Samples of maternal blood, breastmilk, and breastfeeding infant’s blood will be obtained at specific time points for bioanalysis of drug concentrations. Pharmacokinetic data will be analyzed using a population pharmacokinetic approach. Additionally, the study will assess the psychological status of breastfeeding women and the well-being of their infants. Maternal depression is linked to long-term negative consequences for the infant’s physiological regulation, poor growth-promoting setting for the infants, and inappropriate interactive conduct, characterized by low compassion, constrained range of emotional expression, and varying provision of the infant’s budding engagement.

**Conclusions:**

This study will provide the first systematic characterization of mother-to-infant transfer of first-line anti-tuberculosis medicines through breast milk. A mathematical pharmacokinetics model characterizing plasma-to-breastmilk transfer of rifampicin, isoniazid, ethambutol, and pyrazinamide will be developed and used to characterize infant exposure through breast milk. Our findings will contribute towards treatment optimization in breastfeeding and provide a framework to foster other lactation pharmacokinetic studies.

## Introduction

Approximately 50% of women take medication during breastfeeding ^1^. Data regarding the exposure of the breastfed infant to drugs and any associated risks are sparse ^2^. Drugs taken by the breastfeeding mother on tuberculosis (TB) treatment can be passed from the maternal circulation through her breastmilk to the breastfed infant ^3^. This may cause effects on the infant ^4^. Most TB drugs are metabolized by the liver, triggering a potential risk of drug accumulation in infants due to their immature liver function, particularly in premature infants ^5, 6^.

Most drugs are transferred to breastmilk in small quantities, and many have been used without obvious infant toxicity for many years ^3^. Whilst data on TB drug penetration into breast milk is limited, information on clinically relevant infant exposure to these drugs is even more limited. This is an important information gap both for safety, and because therapeutic concentrations could be 1) protective in exposed infants, obviating the need for TB preventive therapy or 2) sub-therapeutic concentrations could select for resistance in those infants infected with Mycobacterium tuberculosis.

Anxiety in mothers, especially those with very young children is not uncommon. Caring for an infant can cause apprehension. This often comes from the pressure to get everything right for the baby. In extreme cases, mothers might suffer postpartum depression. Therefore, the need to take medication during this period (for whatever condition) can aggravate an already fragile mental health state. The association between tuberculosis and depression is well established in non-pregnant adults, including in east African populations ^7, 8^. Depression can be associated with poorer adherence to medication, and therefore with worse treatment outcomes ^9^, hence the need to explore the mental health status of a breastfeeding women.

In this observational study, we aim to characterize the breastmilk transfer of first-line antituberculosis drugs (rifampicin, isoniazid, ethambutol, and pyrazinamide) to the breastfed infant. In addition, we will evaluate the mental health of the breastfeeding mothers enrolled using the Generalised Anxiety Disorder Questionnaire (GAD-7), and Patient Health Questionnaire (PHQ-9), and the well-being of their infants will be assessed using the infant Gross Motor Development (GMD) checklist. Data from this protocol will be pooled and analyzed with data from two other protocols which share standardized methods, and are being conducted within the overarching MILK (Mother-to-Infant Lactation pharmacokinetic) program. Of the two protocols from which we will pool data, one is investigating mothers receiving treatment for uncomplicated malaria in Uganda ^10^ while another is investigating those on drug-resistant TB treatment in South Africa. This collective analysis will allow the exploration of beliefs and attitudes surrounding a range of medication use in breastfeeding mothers.

## Protocol

### Disease setting/patient population

Breastfeeding women receiving first-line antituberculosis treatment and their breastfed infants, including those living with HIV, will be recruited prospectively from the Infectious Diseases Institute (IDI) clinic, and IDI-affiliated Kampala City Council Authority (KCCA) clinics, all in Kampala, Uganda. KCCA clinics are government-funded health facilities that provide a wide range of services, including tuberculosis diagnosis and treatment to the general population, including pregnant and breastfeeding women. We anticipate that 60–80% of women requiring TB treatment will be living with HIV. Drug-drug interactions with antiretroviral therapy (ART) could affect the exposure of TB drugs. The intercurrence of HIV and concomitant antiretroviral medication will be considered as a covariate in the pharmacokinetic analysis. Data on concomitant medication will be recorded and included as a covariate in the pharmacokinetic analysis. The commonly used Antiretroviral therapy (ART) could affect the pharmacokinetic exposure of the TB drugs. The information on the type and dose of ART drugs co-administered to a TB patient will be recorded and integrated into the analysis of the pharmacokinetics of TB drugs to characterise the differences in pharmacokinetic exposure between patients on concomitant TB / ART treatment and those receiving only TB drug. Following the national protocol, infants of mothers diagnosed with DS-TB and treated will receive isoniazid preventive therapy for six months (the duration of the mothers’ tuberculosis treatment).

### Study objectives

***Primary***

1. To characterise the transfer of rifampicin, isoniazid, ethambutol and pyrazinamide to the breastfed infant.
2. To determine the area under the concentration-time curve (AUC), clearance and volume of distribution of these drugs.

***Secondary***

1. To describe covariates influencing drug exposure in maternal plasma, breastmilk and infant plasma
2. To develop a population pharmacokinetic model including the breastmilk and the infant as compartments, which will both enable optimal use of sparse data from future studies, and also enable simulations of different doses or combinations.
3. To assess depression and anxiety levels among breastfeeding mothers on first-line anti-TB drugs.
4. To assess beliefs about medicines in breastfeeding mothers receiving TB treatment.

### Study endpoints

***Primary endpoints***

1. Concentrations of TB drugs in maternal plasma and breastmilk at pre-dose, 2, 4, 6, 8 and, in some cases, 24 hours post-dose.
2. Concentrations of drugs in infant blood at maternal pre-dose, and up to 8 hours post-maternal dose.
3. Area under the concentration-time curve (AUC) of TB drugs in maternal plasma and breastmilk.
4. Breastmilk-to-maternal plasma (M:P) ratio of TB drugs.

***Secondary endpoints***

1. Maximum concentration (Cmax) and time to maximum concentration (Tmax) of TB drugs in maternal plasma and breastmilk.
2. Infant development (using Gross Motor Development score).
3. Depression and anxiety assessments for breastfeeding mothers.
4. Beliefs about medicines in breastfeeding mothers receiving TB treatment.

### Study design

20 Pregnant or lactating women requiring, or those who started, treatment for drug-sensitive tuberculosis will be identified and invited for sampling. If they are pregnant when identified, they will be invited for sampling after delivery. Participants will be identified from recruitment sites by the healthcare unit’s study contact personnel. Plasma and breastmilk samples will be obtained pre-dose and at 2, 4, 6, and 8 hours post-dose. If logistics permit (for example living close to the research unit), participants will be invited for a further sample 24 hours post-dose. A heel prick sample will also be obtained from their breastfed infants at the maternal trough (prior to maternal dose) and at a random time point (once per infant) over the 8-hour pharmacokinetic sampling visit to characterize concentrations of these drugs over an 8-hour dosing interval. The total plasma and breastmilk concentrations of rifampicin, isoniazid, ethambutol, and pyrazinamide will be quantified using liquid chromatography mass spectrometry (LCMS). If a participant has her first pharmacokinetic profile in the intensive phase of TB treatment, (the intensive phase is two months of TB treatment where individuals take all four drugs, namely; rifampicin, isoniazid, ethambutol, and pyrazinamide), she will be invited for a subsequent sampling day with the same time points when she is on the continuation phase of therapy (rifampicin and isoniazid).

### Participant selection

***Inclusion criteria***

Participants must meet all the following inclusion criteria to be eligible for enrollment in the study.

1.    Participants who are willing and able to comply with scheduled visits, treatment plan, laboratory tests, and other study procedures.

2.    A woman is aged 18 years or older.

3.    Receiving treatment for drug-sensitive tuberculosis.

4.    Pregnant or breastfeeding at enrolment.

5.    A personally signed and dated informed consent document indicating that the participant has been informed of all pertinent aspects of the study.

***Exclusion criteria***

Participants presenting with any of the following will not be included in the study:

1.    Severe maternal or infant illness which in the opinion of the patient’s clinician would interfere with her participation in the study.

2.    Breastfed infant is aged over 12 months.

### Treatments of participants, drug storage and drug accountability

All women (and infants) will continue to receive their tuberculosis treatment as prescribed by the physician from the respective TB clinical care sites. Drugs will be stored and dispensed from the relevant TB clinic pharmacy, with no special procedures relating to this observational protocol. The sample size will cater for all the drug categories. First-line tuberculosis treatment consists of rifampicin, isoniazid, ethambutol and pyrazinamide administered as a fixed-dose combination pill. Concomitant medications will be documented on the (case report form) at every study visit.

### Study procedures

***Informed consent***

Potential participants will be identified from the tuberculosis clinic at the IDI and KCCA clinics in Uganda. Informed consent will be obtained for women who express interest in study participation.

All eligible participants must sign an informed consent form before the conduct of any screening procedures. Participants will be encouraged to ask any inquiries concerning the study at this stage.

An impartial witness will be present during the informed consent discussion for participants that are unable to read or write. The witness should be able to read the consent form and participant information leaflet in the participant’s chosen language. After the written, informed consent form is read and explained to the participant, and after they have orally consented to their participation in the study and have either signed the consent form or provided their fingerprint, the witness will sign and personally date the consent form. By signing the consent form, the witness attests that the information in the consent form and any other written information was accurately explained to, and apparently understood by, the participant and that informed consent was freely given by the participant.

***Screening***

**Figure 1.**
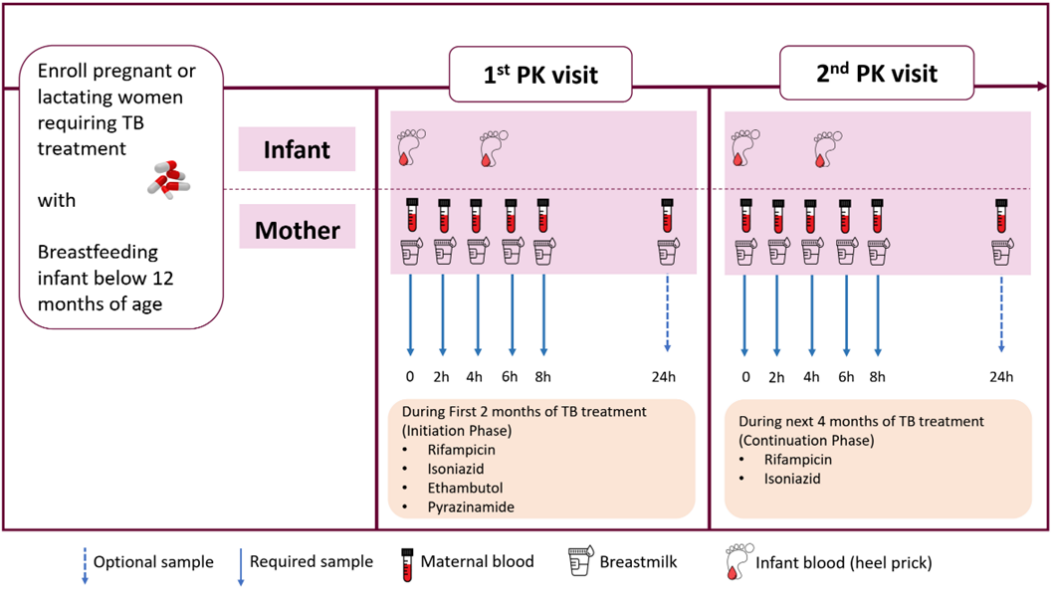
Graphic representation of Pharmacokinetics procedures during the study period.

Participants will be assessed against the eligibility criteria. Some women who intend to breastfeed will be identified in late pregnancy but will still be on TB treatment during breastfeeding. In this situation, consent will be sought, and details of how to contact her will be recorded with the aim to bring her for the pharmacokinetic sampling detailed in Figure 1, at approximately 6 weeks postpartum. The exception to this will be when a mother switches from the four-drug intensive to the two-drug continuation phase of TB treatment before 6 weeks postpartum, in which case attempts to schedule the sampling visit whilst still on the intensive phase will be made.

***Pharmacokinetic study day***

An intravenous cannula will be inserted into her antecubital fossa, and samples taken for trough (pre-dose) drug measurement. After a standardized breakfast, she will be administered her medication. Blood samples will be collected at 2, 4, 6, 8 and ideally* 24 hours post-dose. She will be advised to freely breastfeed her baby. She will be asked to provide a 2–5 ml sample of expressed breastmilk pre-dose, and at 2, 4, 6- and 8-hours post-dosing. A blood sample from the infant will be collected at maternal trough (pre-dose) and at a 3–8 hours post-maternal dose (the second time point will be allocated sequentially to ensure a spread of data points). The mother will be administered a standard lunch as detailed in Table 1.

**Table 1.** Schema for Pharmacokinetic sampling.

		Study Period (8hr) ^, time relative to maternal dose						
Subject	Study Procedure	On arrival	0h	2h	4h	6h	8h	24h ^^^
Mother	Confirm willingness to proceed	x						
	Clinical assessment	x						
	BMI	x						
	Blood for creatinine and Albumin			x				
	Blood PK sample	x		x	x	x	x	x
	BM PK sample	x		x	x	x	x	x
	Standard meal		x			x		
	Observed dosing		x					
	PHQ9, GAD7, BMQ questionnaires				x ˜			
Infant	Clinical assessment	x						
	Weight	x						
	PK sample	x				X *		

*Time of second infant DBS to be recorded, between 3h to 8h ^^^Due to logistic constraints, the 24h may not be collected in every participant ^˜^Can be any time during PK Day

*Due to the logistic considerations of sampling a postpartum mother and her infant who may have travelled a long distance to the clinic, the 24-hour sample may not be collected in all cases. 

Maternal albumin and creatinine will be sampled as they are important for isoniazid exposure. Maternal questionnaires will be filled out on each visit to assess depression and anxiety; Generalised Anxiety Disorder questionnaire (GAD-7), Patient Health Questionnaire (PHQ-9), and the Beliefs about Medicines Questionnaire (BMQ). Infant clinical assessment will include the use of the Gross Motor Development (GMD) checklist (Appendix 1,2, and 3 in the protocol).

### End of TB treatment outcomes

End of TB treatment outcomes (cured, treatment completed, died, defaulted or transferred out) will be collected from the TB registry.

### Participant withdrawal

Participants who may not comply with the protocol’s required schedule of study visits or procedures will be withdrawn from the study. Also, participants may withdraw from the study at any time at their request, or they may be withdrawn at any time at the discretion of the investigator or sponsor for safety or behavioral reasons.

For participants who may miss any study visits (for example, has given consent but then does not return for the pharmacokinetic sampling day detailed in Figure 1), every effort will be made to contact them using telephone numbers documented at enrollment. The investigator will inquire about the reason for withdrawal and follow up with the participant regarding any unresolved adverse events (AEs).

No further evaluations will be performed, and no additional data should be collected if a participant withdraws from the study, and also withdraws consent for disclosure of future information. 

### Assessments

***Safety***

We do not have concerns about the safety of the medication received since this is an observational study. However, adverse events related to the study procedures will be recorded in case report forms and responded to until the event has resolved. Any events that do occur will be listed and analyzed according to Division of Acquired Immune Deficiency Syndrome (DAIDS) criteria that provides an adverse event (AE) severity grading scale. There is no formal requirement for notification of adverse events to the regulatory authorities given that anti-tuberculosis drugs given are standard of care treatment and not an investigational product.

***Pharmacokinetic assessments***

**Blood for pharmacokinetic analysis**

Pharmacokinetic samples will be obtained at the scheduled time relative to dosing and missed sample time will be re-scheduled.

**Sample handling**

Maternal plasma and breast milk will initially be processed and stored in a -80° C at the IDI core laboratory before analysis. The quantification of drug concentrations in maternal and infant plasma and maternal breastmilk will be performed at the IDI core laboratory, or at the pharmacology specialty laboratory at the Division of Clinical Pharmacology, University of Cape Town (UCT) using liquid chromatography-tandem mass spectrometry.

### Validation of breastmilk assay/donor breastmilk

Bioanalysis will employ a liquid chromatography-tandem mass spectrometry (LC-MS/MS) approach which must be validated separately in plasma and breastmilk. It is essential that the standards made to form the standard curve and the different levels of quality control (QC) are spiked into the matrix (plasma, milk, urine etc.) which will be used for the clinical samples. Given there is not yet a fully functioning donor human milk bank in Uganda, we will invite breastfeeding women who are not taking any medication to donate a small amount of breast milk for scientific reasons. These are likely to be staff and students working within IDI, and this approach is frequently used in other centres for the donation of ‘blank’ blood for assay validation. Given that these donors will be drawn from staff and students within the organization, the consent form has not been translated from English.

### Data analysis/statistical methods

***Sample size determination***

This study is exploratory, as no prior study has characterized the exposure of these drugs in maternal plasma, breastmilk and infant plasma. There are no prior data upon which to build a sample size calculation, and there is no comparison between groups which requires statistical analysis with a pre-specified certainty. Since no information is available about the penetration of these drugs into breastmilk, we used the following approach, described in detail for rifampicin.

We modified a previously published population-pharmacokinetic model of rifampicin in plasma, adding a compartment to describe breastmilk concentrations. This was characterised using an approach similar to an effect compartment ^11^ described by a time delay and an accumulation ratio between breastmilk and plasma. The half-life of the delay was fixed to 1 hour and the accumulation ratio to 1.5, with 30% between-subject variability in both parameters. These were chosen to mimic a pharmacokinetic profile similar to Waitt *et al*. ^12^. We assumed 15 individuals (considering a mother-infant dyad as a single unit) with an intensive pharmacokinetic sampling at 0, 1, 2, 4, 6 and 8 hours post-dose of paired plasma and breastmilk (30% error in the breastmilk measurements was assumed) and performed Stochastic Simulations and Estimations (SSEs) ^13^ to evaluate the trial design. Our design can characterise all the typical values of the plasma pharmacokinetic parameters with precision of better than 11% RSE, and all the breastmilk parameters are well characterised with a precision of 1.14% and 0.591% RSE on delay and accumulation ratio, respectively.

An interim analysis will be performed based on the first five participants recruited in this study to assess the adequacy of the sampling design and the bioanalytical methodology in characterising drug concentrations across time.

### Analysis of endpoints

Pharmacokinetic data will be analyzed using a population pharmacokinetic approach to estimate pharmacokinetic parameters and produce modelled fits to exposure data ^14, 15^. Inter-individual variability will be quantified and different covariates tested.

Non-compartmental methods will be used to assess correlations between maternal breast milk drug concentrations and measures of drug exposure in the infant (e.g. AUC) and pharmacodynamic factors.

***Analysis of primary endpoint***

**Non-compartmental analysis**

Plasma pharmacokinetic parameters including the maximum plasma concentration (C _max_), time to maximum plasma concentration (T _max_), and area under the plasma concentration versus time curve (AUC _last_, AUC _τ_) for (drugs) will be estimated using non-compartmental analysis. If data permit or if considered appropriate, area under the plasma concentration versus time curve to infinity (AUC _inf_), terminal elimination half-life (t _1/2_), plasma clearance (CL or CL/F), apparent volume of distribution (V _d_ or V _d_/F) will be also estimated, as data allow. The single dose and/or steady-state PK parameters will be summarized descriptively by dose, cycle and day.

**Population pharmacokinetic (pop-PK) analysis**

The population pharmacokinetic models will be applied to evaluate whether the drug concentrations are consistent with the expected exposures previously reported. If discrepancies between the observed exposure and the model-expected levels are observed, the models will be adjusted by re-estimating the values of the pharmacokinetic parameter values, with the use of Bayesian priors.

Drug concentrations in breastmilk will be linked in the maternal population pharmacokinetic models using an effect compartment strategy, which will allow accurate estimation of the accumulation in breastmilk compared to plasma. Exposure to isoniazid and rifampicin in breastfed infants will be compared with the range of concentrations achieved in adults given therapeutic doses, assuming a minimal duration of exposure from delivery and a maximal duration from the time the mother started the drug (to account for placental transfer).

**Analysis of secondary endpoints**

The data derived from the Generalised Anxiety Disorder questionnaire (GAD-7), Patient Health Questionnaire (PHQ-9), the Beliefs about Medicines Questionnaire (BMQ) and the Infant Gross Motor Development (GMD) checklist will be analysed together with data from the other protocols within the overarching MILK fellowship (mothers on first line malaria treatment in Uganda and on drug-resistant TB treatment in South Africa) and a broader analysis of medication use in breastfeeding. This combined analysis will allow the exploration of beliefs and attitudes surrounding a range of medications used in breastfeeding mothers.

**Safety analysis**

Adverse events relating to the study procedures will be categorized and analyzed.

### Quality control and quality assurance

During study conduct, periodic monitoring may be conducted to ensure that the protocol and good clinical practices (GCPs) are being followed. The monitors may review source documents to confirm that the data recorded on CRFs is accurate. Additionally, the study site may be subject to review by the institutional review board (IRB) and/or to inspection by appropriate regulatory authorities.

### Data handling/record retention

***Case report forms (CRF)***

A CRF is required and should be completed for each included participant.

The investigator has ultimate responsibility for the collection and reporting of all clinical, safety and laboratory data entered on the CRFs and any other data collection forms (source documents) and ensuring that they are accurate, authentic/original, attributable, complete, consistent, legible, timely (contemporaneous), enduring and available when required. The CRFs must be signed by the investigator or by an authorized staff member to attest that the data contained on the CRFs is true. Any corrections to entries made in the CRFs in REDCap (Research Electronic Data Capture) database, source documents must be dated, initialed and explained (if necessary) and should not obscure the original entry.

***Record retention and archiving***

To enable evaluations and/or audits, the investigator agrees to keep records, including the identity of all participating patients (sufficient information to link records, e.g., CRFs and clinic records), all original signed informed consent documents, soft copies of all CRFs, safety reporting forms, source documents, and detailed records of treatment disposition, and adequate documentation of relevant correspondence (e.g., letters, meeting minutes, telephone calls reports).

Investigator records must be kept for as long as required by applicable local regulations (10 years in Uganda).

### Confidentiality and insurance

Clinical data will be entered into a study-specific database by designated staff on a regular basis from completed case record forms (CRF). Case record forms and other source documents will be kept in locked cabinets. Data will be entered on a regular basis to ensure that it is up to date. The database will be entered on regular basis on a secure computer, as will the pharmacokinetic data that will be received by the laboratories. Access to database will be given to authorized personnel only (members of the immediate study team) and a log of authorized personnel will be stored in the trial master file. CRF and trial documents will be kept in locked cabinets. No participant identifying information will be disclosed in any publication or in any conference activities arising from the study.

### Institutional Review Board (IRB)

The protocol, protocol amendments, informed consent documents, and other relevant documents must be approved by the IRB. All correspondence with the IRB should be retained in the regulatory or trial master file. Copies of IRB approvals should be filed with other study documents. The study was reviewed and approved by the Infectious Diseases Institute (IDI) Regulatory Ethics Committee (REC) REF: IDI REC 039/2021 on 06 December 2021, and the Uganda National Council of Science and Technology (UNCST): HS1948ES on 18 January 2023.

### Ethical conduct of the study

The study will be conducted in accordance with legal and regulatory requirements, as well as the general principles set forth in the International Ethical Guidelines for Biomedical Research Involving Human Participants and the Declaration of Helsinki. In addition, the study will be conducted in accordance with the protocol, GCP guidelines, and applicable local regulatory requirements and laws

### Participant information and consent

All parties will ensure protection of participant personal data and will not include participant names on any forms, reports, publications, or in any other disclosures, except where required by law. The informed consent document used in this study, and any changes made during the course of the study, must be prospectively approved by the IRB. The investigator, or a person designated by the investigator, will obtain written informed consent from each participant or the participant's legal representative before any study-specific activity is performed. The investigator will retain the original of each participant's signed consent document.

### Definition of end of study

This will occur when the study enrolls 20 mother infant pairs, and data analysis is complete.

### Publication and dissemination of study results

Study findings will be disseminated to researchers at scientific conferences and peer-reviewed infectious disease and pharmacology journals. Specifically, for Uganda, study results will be communicated to the National Tuberculosis and Leprosy programme in the National Department of Health through meetings and reports. The Community Engagement Plan will detail the engagement with communities at all stages of the protocol from inception through dissemination, and activities under the related Wellcome Public Engagement Enrichment Award Attaining Equity of Access to Research (At The EQUATOR) will ensure meaningful two-way dialogue throughout. Specifically, through At The EQUATOR, the following activities are proposed with regard to the MILK protocols.

### During study set-up

Early consultation with community advisory board (CAB) to determine which community members to approach for dialogue. Initial community meeting with religious leaders, women’s local representative and community members, involving oral discussion and written flyers with pictures and simplified, translated text. Identification of key community members who are interested in co-creation of materials and resources.

### At site initiation

Community meetings will be held at site for Q&A and more specific information about recruitment, and radio broadcast or presentation of drama and song relating to activities will be conducted. Tailor-made flyers and posters including photos with key, simplified text will be shared.

### Dissemination meetings/events

Study progress and results will be conducted and shared with stakeholders.

## Discussion and conclusions

This protocol has been designed with the aim to determine the pharmacokinetics of drugs used to treat drug-sensitive tuberculosis in breastmilk and describe their transfer to a breastfed infant. The current situation where there are no data describing this transfer for rifampicin, ethambutol and pyrazinamide is unacceptable.

Understanding infant exposure to anti-tuberculosis drugs is important to inform on potential risks and benefits to the infant. Measuring drug concentrations directly in the infant is the best way to fully understand of infant exposure and enable informed prediction of infant exposure. Many existing lactation pharmacokinetic studies have estimated maternal-to-infant transfer of drugs (infant exposure) from only maternal plasma and breastmilk drug concentrations. Many authors cite ethical and logistical considerations as reasons for not performing infant blood sampling. However, over the past eight years at the IDI, we have sampled more than 350 mother-infant pairs without encountering such difficulties ^12, 16, 17^). Examples of drugs samples for at IDI include: Tenofovir, Lamivudine, Efavirenz, and Delutegravir.

We have proposed a model-based population pharmacokinetics analysis approach for characterization of changes in drug concentrations across time. Population analysis will enable joint analysis of data from multiple participants, describing both the typical population- and individual-specific profiles. Model-based population analysis offers several advantages over the more commonly used non-compartmental analysis: it can be used for predictions and simulations and for intensive and sparse sampling. We anticipate that findings from this research will enable a clear description of optimal use of drug-sensitive tuberculosis drugs in breastfeeding mothers, will enable development of clear patient information materials and will provide a framework to foster other pharmacokinetic lactation studies in different disease areas. 

## Study status

The study has attained 50% recruitment of the target study sample size.

## Data Availability

No underlying data are associated with this protocol. Zenodo: Pharmacokinetics of drugs used to treat drug sensitive-tuberculosis in breastfeeding mother-infant pairs: An observational pharmacokinetic study. https://doi.org/10.5281/zenodo.7798348 ^18^ Data are available under the terms of the Creative Commons Attribution 4.0 International license (CC-BY 4.0).
