# Supplementary material for: Pharmacokinetics of drugs used to treat drug sensitive-tuberculosis in breastfeeding mother-infant pairs: An observational pharmacokinetic study
Source: Wellcome Open Res. 2024 Aug 12;8:216. Originally published 2023 May 12. [Version 4] doi: 10.12688/wellcomeopenres.19113.4 (PMC11222773; doi:10.12688/wellcomeopenres.19113.4)
Supplement: Peer review history

# Reviewer report: Reviewer response for version 4

**DOI:** 10.21956/wellcomeopenres.25267.r113003

**Published:** 2024-12-04

**Authors:** Gudisa Bereda

**Affiliations:** 1 Pharmacy, All Africa Leprosy Tuberculosis and Rehabilitation Training Centre, Addis Ababa, Addis Ababa, Ethiopia

1. To evaluate covariates influencing drug exposure in maternal plasma, breastmilk, and infant plasma. Discuss the covariates that cause drug exposure in maternal plasma, breastmilk, and infant plasma.

 2. To assess depression and anxiety levels among breastfeeding mothers on first-line anti-TB drugs. Why are depression and anxiety level assessments more critical than other assessments?

 3. To assess beliefs about medicines in breastfeeding mothers receiving TB treatment. How do you assess the beliefs of breastfeeding mothers regarding drugs? Explain the techniques you use to assess beliefs.

 4. Breastmilk-to-maternal plasma (M:P) ratio of TB drugs. What is the ratio of M:P first-line anti-tuberculosis drugs?

 5. Maximum concentration (Cmax) and time to maximum concentration (Tmax) of TB drugs in maternal plasma and breastmilk. What are the Cmax and Tmax of first-line anti-tuberculosis drugs in maternal plasma and breastmilk

 6. Infant development (using the Gross Motor Development score). Why is GMDS assessment required for this study protocol.

Is the study design appropriate for the research question?

Partly

Is the rationale for, and objectives of, the study clearly described?

Yes

Are sufficient details of the methods provided to allow replication by others?

Partly

Are the datasets clearly presented in a useable and accessible format?

Not applicable

Reviewer Expertise:

Infectious diseases, public health, and health related fields

I confirm that I have read this submission and believe that I have an appropriate level of expertise to confirm that it is of an acceptable scientific standard, however I have significant reservations, as outlined above.

# Reviewer report: Reviewer response for version 4

**DOI:** 10.21956/wellcomeopenres.25267.r93303

**Published:** 2024-10-02

**Authors:** Eric Decloedt

**Affiliations:** 1 Stellenbosch University, Stellenbosch, South Africa

Thank you for responding comprehensively to my comments. I am satisfied with the responses.

Is the study design appropriate for the research question?

Yes

Is the rationale for, and objectives of, the study clearly described?

Yes

Are sufficient details of the methods provided to allow replication by others?

Partly

Are the datasets clearly presented in a useable and accessible format?

Not applicable

Reviewer Expertise:

Clinical pharmacology; Pharmacokinetics; HIV-associated comorbidity

I confirm that I have read this submission and believe that I have an appropriate level of expertise to confirm that it is of an acceptable scientific standard.

# Reviewer report: Reviewer response for version 3

**DOI:** 10.21956/wellcomeopenres.23911.r84001

**Published:** 2024-07-03

**Authors:** Belen P. Solans

**Affiliations:** 1 University of California, Los Angeles, USA

No further comments, previous comments should be revised

Is the study design appropriate for the research question?

Yes

Is the rationale for, and objectives of, the study clearly described?

Partly

Are sufficient details of the methods provided to allow replication by others?

Yes

Are the datasets clearly presented in a useable and accessible format?

Not applicable

Reviewer Expertise:

Pharmacokinetics and pharmacodynamics model development in the context of paediatric infectious diseases

I confirm that I have read this submission and believe that I have an appropriate level of expertise to confirm that it is of an acceptable scientific standard, however I have significant reservations, as outlined above.

# Reviewer report: Reviewer response for version 3

**DOI:** 10.21956/wellcomeopenres.23911.r85075

**Published:** 2024-06-18

**Authors:** Eric Decloedt

**Affiliations:** 1 Stellenbosch University, Stellenbosch, South Africa

This is a succinct well-written protocol and the study should make a meaningful contribution to science.

 The protocol will benefit from clarifications of the following points:

- The current inclusion criteria states “Pregnant or breastfeeding at enrolment”. Maternal participants will therefore be at various stages of pregnancy and time post-partum. Some may be 2 ^nd^ trimester, or 3rd trimester with varying durations post-delivery. However, the study objectives do not include pregnancy as a population of interest, but rather focus on breastfeeding mothers (IMPAACT P1026s recently published the PK of RHZE during pregnancy and post-partum [Van Schalkwyk M, et.al., 2023 (Ref 1)]). Will mothers be enrolled during pregnancy but only sampled post-partum? This is also not clear from Figure 1. My impression is that the focus is exclusively on breastfeeding mothers.
- What is the justification for the repeat PK sampling of continuation phase given that similar drugs (RIF & INH) will be sampled? Or do the investigators expect further maternal physiological changes beyond the first 6 weeks post-partum to take place? Explain the justification.
- The NCA analysis plan states that the PK parameter will be summarised descriptively by dose, cycle and day. What cycles and days are referred to? Intensive and continuation phase? Kindly clarify.
- A comment: The sample size for the mental health secondary objective is small and may suffer from recruitment bias. However, the data collected are potentially important for further studies and really only explorative. I am also interested in whether INH exposure (slow acetylator status) is associated with poorer mental health outcomes – beyond the scope of this project.
- To be addressed: Of importance would be a statement/data that the questionnaires GAD-7, PHQ-9 etc have been validated in this population.
- There is little detail on how the outcome “Beliefs about medicines in breastfeeding mothers receiving TB treatment” will be assessed.

Is the study design appropriate for the research question?

Yes

Is the rationale for, and objectives of, the study clearly described?

Yes

Are sufficient details of the methods provided to allow replication by others?

Partly

Are the datasets clearly presented in a useable and accessible format?

Not applicable

Reviewer Expertise:

Clinical pharmacology; Pharmacokinetics; HIV-associated comorbidity

I confirm that I have read this submission and believe that I have an appropriate level of expertise to confirm that it is of an acceptable scientific standard, however I have significant reservations, as outlined above.

## References

[1] : Pharmacokinetics and safety of first-line tuberculosis drugs rifampin, isoniazid, ethambutol, and pyrazinamide during pregnancy and postpartum: results from IMPAACT P1026s. *Antimicrob Agents Chemother* .2023;67(11) : 10.1128/aac.00737-23 e0073723 10.1128/aac.00737-23 37882552 PMC10648924

# Response

**Published:** 2024-08-06

**Authors:** RITAH NAKIJOBA

**Affiliations:** Infectious Diseases Institute, Uganda

A point-by-point response to reviewers’ comments to the manuscript, “Pharmacokinetics of drugs used to treat drug sensitive-tuberculosis in breastfeeding mother-infant pairs: an observational pharmacokinetic study”. We appreciate the thorough review(s) and valuable comments from the editor and the reviewers. We have critically assessed the comments and subsequently revised the manuscript, accordingly, were necessary, while discussing other comments for better clarity, as summarized in the table below.

**Comment:** The current inclusion criteria state “Pregnant or breastfeeding at enrolment”. Maternal participants will therefore be at various stages of pregnancy and time post-partum. Some may be 2 ^nd^ trimester, or 3rd trimester with varying durations post-delivery. However, the study objectives do not include pregnancy as a population of interest but rather focus on breastfeeding mothers (IMPAACT P1026s recently published the PK of RHZE during pregnancy and post-partum [Van Schalkwyk M, et.al., 2023 (Ref 1)]). Will mothers be enrolled during pregnancy but only sampled post-partum? This is also not clear from Figure 1. My impression is that the focus is exclusively on breastfeeding mothers.  

**Response:** In Uganda, antenatal visits provide an opportunity to carry out screening for diseases such as HIV, tuberculosis, and other sexually transmitted diseases (since this is routinely done) to ensure the health of the pregnant woman and growing fetus. As such, many pregnant women only find out that they have HIV or tuberculosis during their pregnancy and that is when they start treatment. Therefore, recruiting a pregnant woman on treatment who is soon to deliver and therefore breastfeed her infant would meet our criteria. This statement has been revised in the inclusion criteria section Thank you for your comment. Indeed, women will be enrolled during pregnancy based on the inclusion criteria outlined in section 4.1, ensuring they are still undergoing TB treatment at the time of delivery. The primary focus for pharmacokinetic (PK) sampling is on breastfeeding mothers.

**Comment:** What is the justification for the repeat PK sampling of the continuation phase given that similar drugs (RIF & INH) will be sampled? Or do the investigators expect further maternal physiological changes beyond the first 6 weeks post-partum to take place? Explain the justification.  

**Response:** The justification for repeat pharmacokinetic (PK) sampling during the continuation phase, despite the use of similar drugs (Rifampicin and Isoniazid), lies in the anticipation of further maternal physiological changes beyond the first 6 weeks postpartum. Postpartum, a woman's body undergoes significant physiological changes, including alterations in drug metabolism and clearance. These changes can continue to evolve beyond the immediate postpartum period. Therefore, repeat PK sampling allows for a more comprehensive understanding of how these drugs are processed in the body as they continue to recover and stabilize after childbirth. This can provide critical insights into dosing and safety for breastfeeding mothers and their infants during the continuation phase of TB treatment. There may also be important changes in breastmilk composition over time. Because of the expected high variability in the population, sampling on two distinct occasions from each participant gives an opportunity to rigorously assess not just inter-individual but also intra-individual variability. This statement has been added in the study design section of the protocol.

**Comment: **The NCA analysis plan states that the PK parameter will be summarised descriptively by dose, cycle and day. What cycles and days are referred to? Intensive and continuation phase? Kindly clarify

**Response:** Yes, the cycles refer to the PK sampling times i.e. the intensive versus continuous phase. We have reworded the phrase to say “summarized by treatment phase i.e. intensive versus continuous.  

**Comment: **A comment: The sample size for the mental health secondary objective is small and may suffer from recruitment bias. However, the data collected are potentially important for further studies and only explorative.

**Response:** You are correct that the sample size for the mental health secondary objective is small. However, the data collected from the Generalised Anxiety Disorder Questionnaire (GAD-7), the Patient Health Questionnaire (PHQ-9), the Beliefs about Medicines Questionnaire (BMQ), and the Infant Gross Motor Development (GMD) checklist will be analyzed in conjunction with data from other protocols within the overarching MILK fellowship. This includes mothers on anti-malarial drugs and TB drugs in Uganda (50) and those on drug-resistant TB treatment in South Africa (20), together with a cohort of breastfeeding mothers recruited at the same clinical sites who are not on any medication. This combined analysis will include approximately a total of 294 participants on different forms of treatment and participants who are not taking any medication and will therefore facilitate the exploration of the mental health of breastfeeding women on a variety of medications. This section is included under the analysis of secondary endpoints.

**Comment: **I am also interested in whether INH exposure (slow acetylator status) is associated with poorer mental health outcomes– beyond the scope of this project.

**Response:** This is an interesting hypothesis, and it could be that NAT2 slow acetylator status means that one has higher concentrations of INH and therefore possibly poorer mental health. However, as you have suggested, exploring that hypothesis goes beyond the scope of this body of work. Indeed, slow acetylator status has previously been associated with neurological complications, and may also be associated with poor mental outcomes. Unfortunately, acetylator status was (is) not evaluated as in this work.

**Comment: **To be addressed: of importance would be a statement/data that the questionnaires GAD-7, PHQ-9 etc have been validated in this population.

**Response: **The statement on how the questionnaires were validated has been added in section 6.3 under study procedures. “The questionnaires were administered to a small sample of the breastfeeding women to identify any issues with wording, understanding, or interpretation of the items”. Furthermore, these tools have been used in other trials in pregnant and breastfeeding women in our population, for example, the DolPHIN-2 trial.

**Comment:** There is little detail on how the outcome “Beliefs about medicines in breastfeeding mothers receiving TB treatment” will be assessed.  

**Response:** Beliefs about medicines in breastfeeding mothers receiving TB treatment” will be assessed using the Beliefs about Medicines Questionnaire (BMQ), as stated in section 6.3.2. The BMQ can effectively assess patients' beliefs about their TB treatment, providing valuable insights that can inform interventions to improve adherence and treatment outcomes it has been used already in our setting. The statement added on pharmacokinetic study day procedures.

# Reviewer report: Reviewer response for version 3

**DOI:** 10.21956/wellcomeopenres.23911.r84002

**Published:** 2024-05-28

**Authors:** Fredrick Rosario-Joseph

**Affiliations:** 1 Division of Reproductive Sciences, Department of Obstetrics and Gynecology, University of Colorado Denver, Denver, Colorado, USA

I accept the revised version of the manuscript.

Is the study design appropriate for the research question?

Partly

Is the rationale for, and objectives of, the study clearly described?

No

Are sufficient details of the methods provided to allow replication by others?

No

Are the datasets clearly presented in a useable and accessible format?

Partly

Reviewer Expertise:

Placental -Fetal Programming

I confirm that I have read this submission and believe that I have an appropriate level of expertise to confirm that it is of an acceptable scientific standard.

# Reviewer report: Reviewer response for version 2

**DOI:** 10.21956/wellcomeopenres.23359.r75637

**Published:** 2024-03-13

**Authors:** Belen P. Solans

**Affiliations:** 1 University of California, Los Angeles, USA

The authors have not modified the manuscript based on previous comments, I have no further comments than those previously made

Is the study design appropriate for the research question?

Yes

Is the rationale for, and objectives of, the study clearly described?

Partly

Are sufficient details of the methods provided to allow replication by others?

Yes

Are the datasets clearly presented in a useable and accessible format?

Not applicable

Reviewer Expertise:

Pharmacokinetics and pharmacodynamics model development in the context of paediatric infectious diseases

I confirm that I have read this submission and believe that I have an appropriate level of expertise to state that I do not consider it to be of an acceptable scientific standard, for reasons outlined above.

# Reviewer report: Reviewer response for version 1

**DOI:** 10.21956/wellcomeopenres.21189.r71785

**Published:** 2024-02-27

**Authors:** Belen P. Solans

**Affiliations:** 1 University of California, Los Angeles, USA

This article provides a study protocol for the characterization of the extent of transfer of drug levels into the breast milk. 

 This protocol will benefit from language revision, as some sentences are disconnected and short, and the appropriate use of connectors would help to better understand the problem the researchers are trying to address. 

**Introduction: **

- Second paragraph: references are lacking for the first sentence.
- Third paragraph: is depression consequence of taking the medication? It would seem so for the way its written, but I don't think that is the message the authors want to give. 
- Four paragraph: clearly state here that you will be looking at DS-TB. What other protocols are the researchers referring to here? This seems vague, similar as with "the other data". If this is a protocol, detail should be given in order to appropriately understand what it is to be done. 

**Protocol:**

- There is a high number of women expected to have concomitant infection with HIV. How would be ART handled or managed? Is there any recommended drugs that investigators expect women will be taking? Are there any known DDIs with those drugs and the first line anti-TB drugs? 
- There is a sentence saying: "diagnosed with tuberculosis treatment", this should be diagnosed with DS-TB and treated. 

**Objectives:**

Primary:

1. Here the authors say "characterize the transfer" - I think this would mean something more like characterizing the mechanism of action, whereas what the researchers are going to do here is to characterize the **extent** of the transfer. 
2. Why do your primary objectives only include AUC, CL and V? Probably better to state that the secondary objective would be to determine the PK characteristics of the drugs. Also, why is V especially relevant here? 

Secondary:

1. Describe or characterize?
2. How is primary objective 2 accomplished without this one? Also, for this part, I would suggest removing all referring to compartments etc, just to develop a model 

**Study endpoints:**

- Pre-dose and up to 8h for children is not very well explained. I would suggest to better explain this as done with the mother sampling schedule, especially when later in the protocol it seems that is not "up to 8h" but rather between 3 and 8 h post maternal dose. 

**Study design:**

- Would the authors require a minimum of mothers in a intensive phase to be able to carry on the study?

**Inclusion criteria:**

- Revise language of this, especially in point 1 (which I would move to 5). Should be: participant provides a signed and dated...

**General comments:**

- The administration of 3 doses prior the one in study should be observed somehow, even by directly reporting of the mother - this will help the pop PK analysis (knowing if you can assume steady state or not, etc). 
- Weight of the mother and malnutrition markers for the children should be removed and analyzed in the PK model.
- If you did an SSE you should be able to provide the power you have to characterize the addition of that effect compartment as opposed to not having it to characterize the breast milk transfer. With respect to power, you should say you will not enroll more than 20 and that is the number it should be used as well. 
- "inter-individual variability will be quantified in relation to the covariates". This is not correct, IIV can and should be characterize irrespective of the covariates included. 
- The use of Non-Compartmental Analysis to characterize correlations or AUC is totally inappropriate if you are doing a popPK compartment model, even more so if you say it on the discussion.

Is the study design appropriate for the research question?

Yes

Is the rationale for, and objectives of, the study clearly described?

Partly

Are sufficient details of the methods provided to allow replication by others?

Yes

Are the datasets clearly presented in a useable and accessible format?

Not applicable

Reviewer Expertise:

Pharmacokinetics and pharmacodynamics model development in the context of paediatric infectious diseases

I confirm that I have read this submission and believe that I have an appropriate level of expertise to confirm that it is of an acceptable scientific standard, however I have significant reservations, as outlined above.

# Response

**Published:** 2024-04-15

**Authors:** RITAH NAKIJOBA

**Affiliations:** Infectious Diseases Institute, Uganda

Comment Response:

 This protocol will benefit from language revision, as some sentences are disconnected and short, and the appropriate use of connectors would help to better understand the problem the researchers are trying to address. Thank you so much for that observation, the team has corrected the grammar in the sentences to make the statements clear, however, you could also specify the exact sentences that are not clear.

**Introduction:**

**Second paragraph**: references are lacking for the first sentence.  

 -Reference has been added in the second part of the introduction. Also, the other studies have been mentioned in the last paragraph of the introduction and referenced (7)

**Third paragraph**: is depression a consequence of taking the medication? It would seem so for the way it's written, but I don't think that is the message the authors want to give.  

 -Anxiety in mothers, especially those with very young children is not uncommon. Caring for an infant can cause apprehension. This often comes from the pressure to get everything right for the baby. In extreme cases, mothers might suffer postpartum depression. Therefore, the need to take medication during this period (for whatever condition) can aggravate an already fragile mental health state   Similarly, taking medication in breastfeeding women may lead to mental health challenges such as depression, which is regularly comorbid with anxiety. Depression can be associated with poorer adherence to medication, and therefore with worse treatment outcomes ^6^ , hence the need to explore the mental health status of a breastfeeding women as mentioned in the introduction section of the manuscript.

**Four paragraphs**: clearly state here that you will be looking at DS-TB. What other protocols are the researchers referring to here? This seems vague, similar to "the other data". If this is a protocol, detail should be given to appropriately understand what is to be done.  

 -The other protocols that will collect data on the infant's gross motor development have been included as references  in the last paragraph of the introduction and referenced (7)

**Protocol**: There is a high number of women expected to have a concomitant infection with HIV. How would be ART handled or managed? Is there any recommended drugs that investigators expect women will be taking? Are there any known DDIs with those drugs and the first-line anti-TB drugs?  

 -The Uganda Ministry of Health guidelines recommend the use of triple therapy; Tenofovir, lamivudine, and Dolutegravir as first-line ART drugs. The drugs taken by persons living with HIV will be documented. Studies have used population pharmacokinetic modeling to compare the pharmacokinetics of dolutegravir when co-administered with standard- versus high-dose rifampicin in adults with tuberculosis and HIV, Simulations of trough dolutegravir concentrations show that the 50 mg twice-daily regimen attains both the primary and secondary therapeutic targets of 0.064 and 0.3 mg/L, respectively, regardless of the dose of co-administered rifampicin, unlike the once-daily regimen. We anticipate that 60–80% of women requiring TB treatment will be living with HIV. Drug-drug interactions with antiretroviral therapy (ART) could affect the exposure of TB drugs. The intercurrence of HIV and concomitant antiretroviral medication will be considered as a covariate in the pharmacokinetic analysis, As stated in disease setting and population section. There is a sentence saying: "diagnosed with tuberculosis treatment", this should be diagnosed with DS-TB and treated.   The sentence has been corrected under the disease setting and study population section.

**Objectives:** Primary: Here the authors say "characterize the transfer"- I think this would mean something more like characterizing the mechanism of action, whereas what the researchers are going to do here is to characterize the extent of the transfer. We thank the author for highlighting this possible source of confusion. We will characterize the pharmacokinetic processes associated with the transfer of the four drugs to the breastfed infant.

 -An adjustment has been made “To characterize the pharmacokinetic transfer of rifampicin, isoniazid, ethambutol and pyrazinamide to the breastfed infant” Why do your primary objectives only include AUC, CL and V? Probably better to state that the secondary objective would be to determine the PK characteristics of the drugs. Also, why is V especially relevant here?   We thank the reviewer for raising this comment. We acknowledge that there are several additional PK parameters (Cmax, Tmax..) that could be estimated from this data and indeed will be evaluated in the analysis. AUC in particular provides and overall measure of drug exposure and in lactation pharmacokinetic studies it is widely employed in comparing plasma-to-breast milk transfer of the drug, whereas CL and V are two parameter that closely affect the drug elimination. We have not made changes to the manuscript

**Secondary**: Describe or characterize?  

 -We thank the reviewer for this comment. More appropriately we will evaluate. We have changed secondary objective #1 How is primary objective 2 accomplished without this one? Also, for this part, I would suggest removing all referring to compartments etc, just to develop a model   The primary objectives will be accomplished using non-compartmental analysis. A population pharmacokinetic model will be developed secondarily. The details of the model provides the rational for developing the model, as such it is important to highlight the components of the model. We have edited this objective   To develop a population pharmacokinetic model characterizing the transfer of drugs from maternal plasma to breast milk and resultant infant exposure  

**Study endpoints**: Pre-dose and up to 8 hours for children are not very well explained. I would suggest better explaining this as done with the mother sampling schedule, especially when later in the protocol it seems that is not "up to 8h" but rather between 3 and 8 h post maternal dose.  

 -Thank you for this comment, however as stated in the protocol; A blood sample from the infant will be collected at the maternal trough (pre-dose) and at a 3– 8 hours post-maternal dose (the second time point will be allocated sequentially to ensure a spread of data points). The second time point will be taken at any hour between 3-8 hours post maternal dose, if the mother fails to complete the 8 hours, then the infant will be sampled earlier than the last time point.

**Study design:** Would the authors require a minimum of mothers in an intensive phase to be able to carry on the study?  

 -We need not specify a minimum number of mothers enrolled in the intensive phase however we shall ensure that more than 50% of the mothers are enrolled during the intensive phase.

**Inclusion criteria**: Revise language of this, especially in point 1 (which I would move to 5). Should be: participant provides a signed and dated...  

 -Thank you for this observation, we agree that signing the ICF comes after it has been confirmed that the study participant is eligible for the study. Having it as #5 does not change the fact that it is important criteria   .

**General comments**: The administration of 3 doses before the one in the study should be observed somehow, even by directly reporting to the mother - this will help the pop PK analysis (knowing if you can assume a steady state or not, etc).    

 -You raise a very important point, unfortunately, the study will depend on patient adherence reports and pill count for the last three days before intensive PK sampling day. However, in order to capture this with the greatest degree of accuracy, the study nurses make phone calls to the study participants during the five days prior to the PK sampling day to remind them of the need for good adherence to the medication, and to capture the dosing time. Other procedures such as video DOTS are not possible in our setting because the majority of the study participants do not have access to smartphones. Weight of the mother and malnutrition markers for the children should be removed and analyzed in the PK model.  

 The study captures both weight and nutrition status of the mother and infant. Both variables will be removed and analysed in the model. As stated in analysis of endpoints section;” Inter-individual variability will be quantified and different covariates tested”.       If you did an SSE you should be able to provide the power you have to characterize the addition of that effect compartment as opposed to not having it to characterize the breast milk transfer. With respect to power, you should say you will not enroll more than 20 and that is the number it should be used as well.  

 -The half-life of the delay in maternal plasma-to-breast milk transfer and the accumulation rate constant were both fixed for the SSE.     "inter-individual variability will be quantified in relation to the covariates". This is not correct, IIV can and should be characterize irrespective of the covariates included.  

 -We thank the reviewer for  this point – We acknowledge our wording is confusing and have made amends:   “The impact of covariates on Interindividual variability in pharmacokinetic parameters will be evaluated” The use of Non-Compartmental Analysis to characterize correlations or AUC is totally inappropriate if you are doing a popPK compartment model, even more so if you say it on the discussion.  

 -We thank the reviewer for this point – We acknowledge our wording is confusing. Comparison of exposure in maternal plasma and breast milk and infant exposure will be based on standard statistical tests and not non-compartmental pharmacokinetic analysis

# Reviewer report: Reviewer response for version 1

**DOI:** 10.21956/wellcomeopenres.21189.r59255

**Published:** 2023-09-20

**Authors:** Fredrick Rosario-Joseph

**Affiliations:** 1 Division of Reproductive Sciences, Department of Obstetrics and Gynecology, University of Colorado Denver, Denver, Colorado, USA

The rationale for doing the behavior test needs to be addressed in the background of the abstract.  

 The total study population is n=20; how many n for each drug category (rifampicin, isoniazid, ethambutol, and pyrazinamide)?

 What are the current studies dealing with TB drugs and it is breastfeeding content?

 Does the ethnicity have any impact on it?

 Secondary metabolite levels are not measured? Any impact on the infant?

 It is confusing and unclear suddenly introduce uncomplicated malaria data with TB resistance. What outcome was added to the current data? Be clear in explaining the rationale.  

 Again introducing the HIV-infected group (60-80%) and only the remaining 20% would be noninfected HIV group taking antituberculosis treatment? No possibility of getting the statistical test comparison?  

 What is the Km value of each drug, and what is the half-life? 

 "Antiretroviral therapy (ART) could affect exposure of TB drugs. Data on concomitant medication will be recorded and included as a covariate in the pharmacokinetic analysis" - Elaborate on it.

 How would you separate the indirect absorption rate among each drug group? What analysis will eliminate the cross-over reaction among the drugs?

 "Pregnant or lactating women requiring, or those who started, treatment for drug-sensitive tuberculosis will be identified and invited for sampling". How many n from each group? It isn't very clear with the study design.

 What time points for the predose? 

 Will the weight be normalized to the maternal and infant plasma/breast milk concentration? 

 Is any sex-specific effect on absorption included in the analysis?

Is the study design appropriate for the research question?

Partly

Is the rationale for, and objectives of, the study clearly described?

No

Are sufficient details of the methods provided to allow replication by others?

No

Are the datasets clearly presented in a useable and accessible format?

Partly

Reviewer Expertise:

Placental -Fetal Programming

I confirm that I have read this submission and believe that I have an appropriate level of expertise to state that I do not consider it to be of an acceptable scientific standard, for reasons outlined above.

# Response

**Published:** 2024-02-15

**Authors:** RITAH NAKIJOBA

**Affiliations:** Infectious Diseases Institute, Uganda

**Reviewer 1** **Comment** **Response** The rationale for doing the behavior test needs to be addressed in the background of the abstract.  

   The statement added on rationale for the behavior test in the background section of the abstract.   Similarly, women face mental health challenges while taking medications, including postpartum depression. Specifically, it is recognized that rates of depression are high during treatment for tuberculosis. This may have an impact on their motivation to take their medicine, hence the need to assess the psychological behavior of a breastfeeding woman The total study population is n=20; how many n for each drug category (rifampicin, isoniazid, ethambutol, and pyrazinamide)? The study will recruit 20 breastfeeding women overall. The sample size will cater to all the drug categories. First-line tuberculosis treatment consists of rifampicin, isoniazid, ethambutol, and pyrazinamide administered as a fixed-dose combination pill. This has been specified in the abstract.  More detail is given further in the protocol. women will be recruited regardless of the phase of TB treatment, intensive and continuous phases. This is well documented in the study procedures under the screening section. There has been no strong data upon which to build a sample size calculation. We have discussed the interim analysis to refine some of our assumptions – adding a line to the abstract: “Pharmacokinetic data will be analyzed using a population pharmacokinetic approach, and an interim analysis of rifampicin concentrations will be conducted after the first 5 participants have had a pharmacokinetic sampling.” What are the current studies dealing with TB drugs and it is breastfeeding content?

   No recent studies have investigated TB drugs in breastfeeding women. However, old studies from 1965-1990 explored TB drugs among breastfeeding women including; Ricci and Copaitich reported peak levels of INH in breast milk of 6 mcg/mL and 9 to I I mcg/mL after the administration of 5 mg/kg and 10 mg/kg, respectively, given to three women, Knowles measured milk INH concentrations taken 1—10 hours after the administration of 5—10 mg/kg, Holdiness reported a maximum pyrazinamide breast milk concentration of 1.5 mcg/mL measured 3 hours after maternal ingestion of a single I gram dose as compared to a maternal plasma concentration of 42 mcg/mL 2 hours after the dose,   Does the ethnicity have any impact on it?

           Secondary metabolite levels are not measured? Any impact on the infant?

   In previous studies in non-pregnant, non-breastfeeding adults, ethnicity has not had a significant impact on drug concentrations. For isoniazid, metaboliser status according to the polymorphic NAT enzyme plays a role.    A full investigation of the impact of ethnicity and pharmacogenomic analysis is beyond the scope of this study, although our results may inform the design of a future, larger, multinational investigation.     In this protocol, we will not measure any secondary metabolites in either plasma or breast milk. However, if our study finds that appreciable amounts of the drug end up in breastmilk/infant we can then assume that this might translate to particular metabolites as well.   It is confusing and unclear to suddenly introduce uncomplicated malaria data with TB resistance. What outcome was added to the current data? Be clear in explaining the rationale.  

   This has been clarified to explain that these other studies are sister protocols taking place under the MILK fellowship. The drugs and diseases are different, but we are using the same approach to the mental health aspects of the protocols and therefore, those specific data will be analysed together with the other work. Again, introducing the HIV-infected group (60-80%) and only the remaining 20% would be noninfected HIV group taking anti-tuberculosis treatment? Any possibility of getting the statistical test comparison?

The intercurrence of HIV and concomitant antiretroviral medication will be considered as a covariate in the pharmacokinetic analysis. The statement has been added to the protocol section; Disease setting and patient population What is the Km value of each drug, and what is the half-life?  Generally, high inter-patient variability in pharmacokinetics has been reported for the four first-line anti-tuberculosis drugs. Rifampicin has a time-to-peak plasma concentration of approximately two hours after oral dosing and a half-life of approximately 2.5 hours (doi: 10.2165/00003088-197803020-00002). The plasma half-life of isoniazid is closely related to the patient’s genetics (doi: 10.1128/AAC.02599-12), ranging from approximately one hour in fast acetylators to more than 3 hours in slow acetylators. The peak plasma concentrations of pyrazinamide range from 0.8 – 8 hours after dose, whereas its half-life ranges from 4.5 - 11 hours (doi.org/10.1007/s13318-018-00540-w; https://doi .org/10.1128/AAC.02625-16). The peak plasma concentrations of ethambutol occur 2 - 4 hours after administration and has a plasma half-life between 3-4 hours (doi: https://doi.org/10.1128/aac.01583-19).   " Antiretroviral therapy (ART) could affect exposure of TB drugs. Data on concomitant medication will be recorded and included as a covariate in the pharmacokinetic analysis" - Elaborate on it. " The commonly used Antiretroviral therapy (ART) could affect the pharmacokinetic exposure of the TB drugs. The information on the type and dose of ART drugs co-administered to a TB patient will be recorded and integrated into the analysis of the pharmacokinetics of TB drugs to characterise the differences in pharmacokinetic exposure between patients on concomitant TB / ART treatment and those receiving only TB drug. Statement added How would you separate the indirect absorption rate among each drug group? What analysis will eliminate the cross-over reaction among the drugs?

   Thank you for the question: For clarification, Although TB medication is administered as a fixed dose combination, different assays will be applied to the pharmacokinetic samples to quantify the concentration of each drug for each patient at a given time point. From these concentration-time data, we will develop drug-specific population pharmacokinetic models, one for rifampicin, isoniazid, ethambutol and pyrazinamide. These mathematical models will describe the concentration-time profiles for each drug accounting for the dose administered and estimate different pharmacokinetic parameters. As such the rate and extent of drug absorption will be individually characterised for each of the four drugs basing on the collected pharmacokinetic data. "Pregnant or lactating women requiring, or those who started, treatment for drug-sensitive tuberculosis will be identified and invited for sampling". How many n from each group? It isn't very clear with the study design.

   The target recruitment number of 20 has been added in the study design section to reflect the required sample size. What time points for the predose? 

   The pre-dose sample will be obtained at different time points depending on what time the participant has reported to the clinic. The participant will come to the clinic first thing in the morning before taking their dose of treatment. A pre-dose blood sample will be drawn within 30 minutes of the observed dosing. Will the weight be normalized to the maternal and infant plasma/breast milk concentration? 

   Weight and BMI will both be included as covariates in the pharmacokinetic model. Is any sex-specific effect on absorption included in the analysis? This population is breastfeeding mothers only. It is known that pregnancy may slow drug absorption due to delayed gastric emptying and reduced gastric pH – and some of this change may persist into the postpartum period. The intensive sampling after dose aims to ensure we characterize absorption well, and can then compare with similar work from non-pregnant adult populations, including both males and females.
